# A Decrease in Longitudinal Length of Kidney is a Reliable Tool to Predict the Success of Pyeloplasty in Children

**DOI:** 10.1111/iju.70175

**Published:** 2025-07-11

**Authors:** Dogancan Dorucu, Kader Ada Dogan, Onur Can Ozkan, Cagri Akin Sekerci, Yiloren Tanidir, Tufan Tarcan, Selcuk Yucel

**Affiliations:** ^1^ Department of Urology School of Medicine, Marmara University Istanbul Turkey; ^2^ Division of Pediatric Urology, Department of Urology School of Medicine, Marmara University Istanbul Turkey; ^3^ Medicana Ataşehir Hospital Istanbul Turkey; ^4^ Department of Urology School of Medicine, Koç University Istanbul Turkey

**Keywords:** children, hydronephrosis, kidney diameters, pyeloplasty, ureteropelvic junction obstruction

## Abstract

**Objectives:**

The renal pelvis anteroposterior diameter (RPAPD) is an important parameter used in the indication and follow‐up of ureteropelvic junction obstruction (UPJO). We hypothesized that kidney dimensions, namely longitudinal length (LL) and transverse width (TW), may have an easier similar validity to RPAPD measurement in the diagnosis of UPJO and follow‐up after pyeloplasty.

**Methods:**

Children who underwent pyeloplasty (January 2012–January 2024) were retrospectively evaluated. Exclusion criteria included megaureter, vesicoureteral reflux, urinary stones, duplicated systems, abnormal contralateral kidneys, secondary interventions, and incomplete data. The RPAPD, hydronephrosis grade, LL, and TW measured by ultrasound (US) before and 6 months after pyeloplasty were compared.

**Results:**

Forty‐nine children (14 girls, 35 boys; age range: 6 months to 17 years) who underwent pyeloplasty were studied. A significant reduction in RPAPD (29 to 18 mm) and LL (99 to 95 mm) was observed in affected kidneys 6 months after pyeloplasty compared to preoperative US measurements (*p* < 0.0001 and *p* = 0.005, respectively) but not in TW (*p* = 0.19). Similarly, the ratio of LL of the affected kidney to contralateral kidney (1.2 to 1.12 mm) significantly decreased after pyeloplasty (*p* = 0.026) but not the ratio of TW (*p* = 0.357). A positive correlation between RPAPD and LL is revealed (correlation coefficient = 0.619, *p* < 0.001).

**Conclusions:**

The present study indicates that LL was elevated in affected kidneys compared to contralaterals and significantly decreases after pyeloplasty. We suppose that the decrease in LL may be an alternative, straightforward, and reliable measurement to assist in the follow‐up after pyeloplasty.

## 1 Introduction

Ureteropelvic junction obstruction (UPJO) is the most common pathological cause of hydronephrosis in infants and children [1]. It results in impaired urine drainage into the ureter leading to hydronephrosis. Obstructive state of the UPJ can precipitate a wide range of complications, including flank pain, stone formation, pyelonephritis, and diminished renal function. Pyeloplasty is the main surgical procedure to preserve renal function in cases with failed conservative management [2]. Monitoring the resolution of hydronephrosis is essential following the surgical procedure due to a small but solid failure rate of 2.5%–10% [3, 4].

Hydronephrosis in children is assessed by radiological studies, such as renal ultrasound (US), computerized tomography (CT) or magnetic resonance imaging (MRI), and nuclear dynamic renal scintigraphy with mercaptoacetyltriglycine (MAG3). Various classifications are present to evaluate hydronephrosis, including the Society for Fetal Urology (SFU) grade [5] and Urinary Tract Dilation (UTD) consensus [6]. Also, geometric measurements, such as renal pelvis anteroposterior diameter (RPAPD), renal pelvis volume, and renal cortical thickness (RCT), are used to determine the severity of hydronephrosis. RPAPD measured by US is one of the important tools used in determining the indication for pyeloplasty and also for monitoring the success of intervention. An increased RPAPD may suggest hydronephrosis or obstruction, and there is a moderate correlation between AP diameter on MAG3 scintigraphy and functional obstruction [7]. However, factors such as hydration status and incomplete bladder emptying influence the accuracy of RPAPD measurements on ultrasonography [8].

It is also utilized as a part of scoring systems such as the Pyeloplasty Prediction Score (PPS) and percentage anteroposterior diameter improvement (PI‐APD) [9, 10]. Renal cortical thickness and renal parenchyma‐to‐hydronephrosis area ratio (PHAR) after pyeloplasty are also suggested to demonstrate the success of surgery [11, 12]. To predict the success of pyeloplasty, the change in collecting system circularity and roundness and the changes in ratio of renal parenchymal area to collecting system area (RPCSR) have also been defined [13]. However, these methods are technically and financially demanding due to the requirement of an experienced ultrasound technician and/or radiologist, and moreover, there is substantial disagreement on their interpretation. Therefore, we hypothesized that change in kidney longitudinal and transverse diameters measured by US may be a more straightforward and reliable tool for diagnosis and follow‐up after pyeloplasty.

## 2 Materials and Methods

The protocol for this research project has been approved by a suitably constituted Ethics Committee of Marmara University, and it conforms to the provisions of the Declaration of Helsinki. (Approval No. 09.2024.340). Electronic records of all patients in 0–18 years of age who underwent open or laparoscopic pyeloplasty between January 2012 and January 2024 are studied. Children with megaureter, vesicoureteral reflux (VUR), duplicated system, abnormal contralateral kidney, prior pyeloplasty, and missing or incomplete data are excluded. For each patient, medical history, blood and urine biochemistry tests, urinary US, and MAG3 before and after pyeloplasty were assessed. Open or laparoscopic dismembered pyeloplasty was performed on patients requiring surgical repair. Only the last US before the pyeloplasty and the first US after 6 months of pyeloplasty were compared in the study. We specifically recorded the changes in RPAPD, kidney longitudinal length (LL), and transverse width (TW) before and after surgery. The LL of the kidney is defined as the distance between its superior and inferior poles. The TW is defined as the maximum horizontal distance across the kidney, typically measured in the axial plane. Also, the ratio of affected kidneys to contralateral kidneys for LL and TW was assessed. All ultrasounds were performed by different radiologists within the same department. As in the approach to patients with hydronephrosis, ultrasounds were performed as part of routine preoperative and postoperative follow‐up. Correlation between hydronephrosis grade, RPAPD, LL, and TW was also analyzed. Lastly, preoperative US measurements of kidney and diuretic response in MAG3 were compared.

### 2.1 Statistical Analyses

Data entry and analyses were done using Statistical Package for Social Sciences (SPSS) version 25.0 (IBM Corporation, Armonk, New York). The normality of the distribution of the variables was evaluated using the Shapiro–Wilk test. As the distribution of continuous variables did not show a normal distribution, continuous data were presented with median, minimum, and maximum. Comparisons of independent and dependent groups were done with the Mann–Whitney *U* test and Wilcoxon signed ranks test, respectively. Multiple group analyses were performed with Kruskal–Wallis test. Correlation analysis was done with the Spearman correlation test. *p* value < 0.05 was considered statistically significant.

## 3 Results

A total of 64 patient charts were found, and 49 children (median age of 6 years, range: 4 months to 17 years, 14 girls [28.6%] and 35 boys [71.4%]) were included in the study. Right and left pyeloplasties were performed on 19 (38.8%) and 30 (61.2%) children, respectively. Laparoscopic pyeloplasty was performed in 15 (30.6%) and open pyeloplasty in 34 (69.4%) children.

Preoperative RPAPD, kidney LL, and TW were 29 mm (range: 15–76 mm), 99 mm (range: 72–146 mm), and 38 mm (range: 22–74 mm), respectively. After pyeloplasty, renal pelvis AP diameter, LL, and TW were 18 mm (range: 3–57 mm), 95 mm (57–140 mm), and 37 mm (range: 20–75 mm), respectively. As seen in Table 1, there was a significant decrease in RPAPD and LL in the operated kidney at the 6th month after the pyeloplasty compared to the preoperative US (*p* < 0.0001, *p* = 0.005, respectively). However, no significant difference was observed in TW of the affected kidney after surgery (*p* = 0.19). Preoperatively, the ratio of the ipsilateral to contralateral kidney was 1.2 for LL (range: 0.98–1.77) and 1.15 for TW (range: 0.70–1.68). After surgery, the ratio of the ipsilateral to contralateral kidney was 1.12 for LL (range: 0.73–1.66) and 1.06 for TW (range: 0.68–1.74). We found a significant decrease in the LL ratio, whereas TW ratio did not change significantly (*p* = 0.026, *p* = 0.357, respectively) (Table 1).

**TABLE 1:** Comparison of preoperative and postoperative diameters of the affected kidney.

	Preoperative	Postoperative	*p*
Renal pelvis AP diameter (mm) Median (min–max)	29 (15–76)	18 (3–57)	**< 0.0001**
Longitudinal length (mm) Median (min–max)	99 (72–146)	95 (57–140)	**0.005**
Ratio of ipsilateral/contralateral kidney for longitudinal length Median (min–max)	1.2 (0.98–1.77)	1.12 (0.73–1.66)	**0.026**
Transverse width (mm) Median (min–max)	38 (22–74)	37 (20–75)	0.19
Ratio of ipsilateral/contralateral kidney for transverse width Median (min–max)	1.15 (0.70–1.68)	1.06 (0.68–1.74)	0.357

*Note:* Bold values indicate *P* < 0.05.

As seen in Table 2, preoperative LL and TW were detected in the normal contralateral kidney as 84 mm (range: 55–124 mm) and 36 mm (range: 22–54 mm), respectively. Postoperatively, LL and TW were seen as 84.5 mm (range: 61–129 mm) and 35.5 mm (range: 27–49 mm), respectively. A significant difference in LL was observed between the preoperative ipsilateral and contralateral normal kidney (*p* < 0.0001). Transverse width was not different in both kidneys before and after pyeloplasty (*p* = 0.051, *p* = 0.249, respectively) (Table 2).

**TABLE 2:** Comparison of preoperative and postoperative diameters of ipsilateral and contralateral kidneys.

	Renal diameters	Ipsilateral	Contralateral	*p*
Preoperative	Longitudinal length (mm) Median (min–max)	99 (72–146)	84 (55–124)	**< 0.0001**
	Transverse width (mm) Median (min–max)	38 (22–74)	36 (22–54)	0.051
Postoperative	Longitudinal length (mm) Median (min–max)	95 (57–140)	84.5 (61–129)	**0.003**
	Transverse width (mm) Median (min–max)	37 (20–75)	35.5 (27–49)	0.249

*Note:* Bold values indicate *P* < 0.05.

There are only four patients whose renal pelvis AP diameter has increased or not changed. The LL increased only in 1 of them (110–130 mm), and this one required a secondary intervention in the follow‐up. All other three had either stable or decreased LL in the 6th month US and showed decreasing renal pelvis AP diameters in 6 months after first 6th month US control. In the correlation analysis, a positive correlation was detected between both preoperative renal pelvis AP diameter and LL. Grade of hydronephrosis showed positive correlation with TW. However, no correlation was found between renal pelvis AP diameter and TW (Table 3).

**TABLE 3:** Correlation of preoperative hydronephrosis grade and kidney diameters.

		Hydronephrosis grade	Renal pelvis AP diameter	Longitudinal length	Transverse width
Hydronephrosis grade	Correlation coefficient	1.000	0.140	0.110	**0.344** *
	Sig. (2‐tailed)	.	0.337	0.458	0.018
	*N*	49	49	48	47
Renal AP diameter	Correlation coefficient	0.140	1.000	**0.619** **	0.244
	Sig. (2‐tailed)	0.337	.	0.000	0.098
	*N*	49	49	48	47
Longitudinal length	Correlation coefficient	0.110	**0.619** **	1.000	**0**.**508** **
	Sig. (2‐tailed)	0.458	0.000	.	0.000
	*N*	48	48	48	47
Transverse width	Correlation coefficient	**0.344** *	0.244	**0.508** **	1.000
	Sig. (2‐tailed)	0.018	0.098	0.000	.
	*N*	47	47	47	47

*Note:* Spearman rho.

* Correlation is significant at the 0.05 level (2‐tailed).

** Correlation is significant at the 0.01 level (2‐tailed).

**FIGURE 1:**
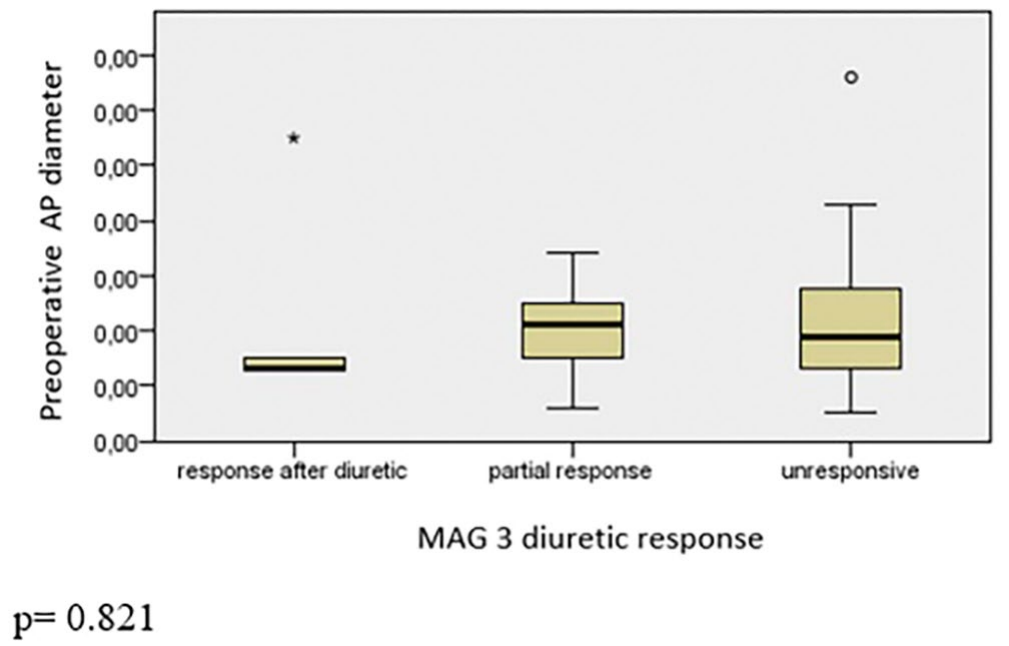
Comparison between diuretic response and preoperative anteroposterior diameter.

**FIGURE 2:**
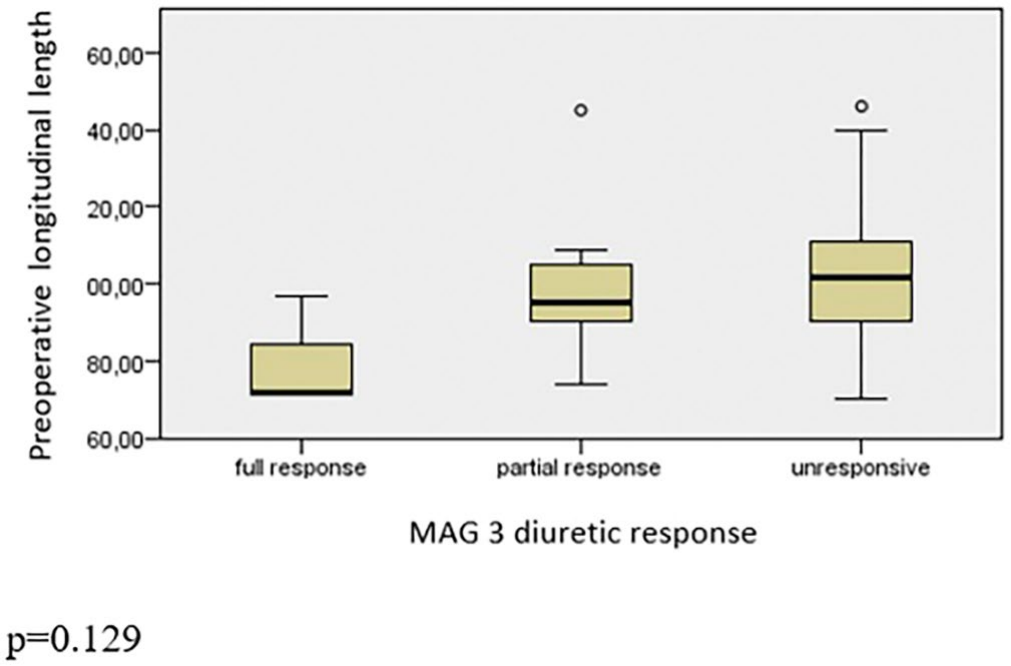
Comparison between diuretic response and preoperative longitudinal length.

**FIGURE 3:**
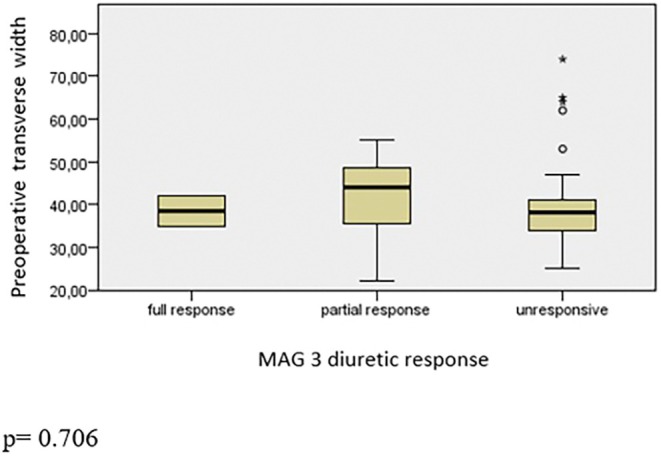
Comparison between diuretic response and preoperative transverse width.

Nuclear dynamic renal scintigraphy showed a median of 43.6% (range: 14%–59%) function of the operated kidneys. Of the 49 children, preoperatively, 3 had a complete response to diuretic, 12 had a partial response, and 34 had no responses. There was no significant difference between responsiveness and RPAPD (*p* = 0.821, Figure 1), LL (*p* = 0.129, Figure 2), and TW (*p* = 0.706, Figure 3). A differential renal function (DRF) below 40% was observed in 11 children (22.4%), while 38 children (77.6%) had a DRF above 40%. No statistically significant differences in LL were observed between the DRF < 40% and DRF > 40% groups in either the preoperative (*p* = 0.078) or postoperative (*p* = 0.088) periods. Additionally, within‐group comparisons revealed no significant change in LL values following surgery. Specifically, in the DRF < 40% group, the difference between preoperative and postoperative measurements did not reach statistical significance (*p* = 0.050). Similarly, in the DRF > 40% group, no significant difference was observed between pre‐ and postoperative values (*p* = 0.108).

## 4 Discussion

In the diagnosis and follow‐up of children with UPJO, US has an important place due to easy access and no radiation exposure. RPAPD, RCT, and hydronephrosis grade are commonly evaluated to classify the state of obstruction and resolution. In this study, we aimed to compare the dimensions of the kidney with the hydronephrosis grade and RPAPD following pyeloplasty as the surgical management to treat UPJO in children. We found that only the LL of the kidney was significantly decreased with a positive correlation to decrease in RPAPD at the first 6 months of pyeloplasty. Interestingly, the TW of the affected kidney was comparable to normal contralateral kidney preoperatively and postoperatively.

To evaluate the success of pyeloplasty, different modalities like renal US, nuclear dynamic renal scintigraphy, CT, MRI, and urinary biomarkers have been utilized. Previous studies point out that renal scintigraphy is the main tool to indicate the necessity of surgical repair, but US is more commonly used due to its less invasive properties [14]. RPAPD has a pivotal role in showing the degree of obstruction and predicting the need for surgical intervention. Arora et al. [15] found that RPAPD of 24 mm could predict the need for surgery, with a sensitivity of 73.1% and a specificity of 88.0%. Also, they showed that RPAPD and differential renal function in renal scintigraphy are the independent predictive factors for surgery. Dias et al. [16] suggest that a cutoff of 16 mm of RPAPD be predictive of surgery with a sensitivity of 100% and specificity of 86%. After surgical intervention, it is expected an early decrease in RPAPD due to relief of obstruction and/or surgical pelvis reduction. However, monitoring of RPAPD can also demonstrate long‐term outcomes [17]. Rickard et al. [10] showed that a less invasive follow‐up may be achieved by using a cutoff value of 40% for the percentage RPAPD improvement. As RPAPD measurement is not standardized [18], there are still some pitfalls that require expertise. Additionally, RPAPD can be affected by many factors such as bladder filling, excessive hydration, and position of the patient [19].

Several studies showed that RCT can be associated with nephron numbers [20]. However, the time to surgical intervention is not associated with the change in RCT. To predict postoperative differential renal function improvement, baseline RCT can be beneficial [21]. Also, Chalieopanyarwong and Attawettayanon [11] found that RCT after surgery was improved in follow‐up, thus proposing an indirect marker of improved renal function and pyeloplasty success. Kern et al. [13] presented that the change in collecting system roundness and the ratio of renal parenchymal to collecting system area were reliable in predicting pyeloplasty success. Rickard et al. [12] established that renal PHAR may help to predict future surgical needs in newborns with high‐grade hydronephrosis. Han et al. [22] also showed the high ratio of hydronephrosis area to renal parenchyma estimated undesirable surgical results. Obviously, those derived measurements and calculations would require a dedicated ultrasound technician and radiologist as well as longer clinical time periods.

The measurement of RPAPD is an operator‐dependent technique that requires experience. Furthermore, it is controversial whether optimal measurement should be done at the renal contour or the inner part of the pelvis. We believe that more reliable and less time and expertise consuming measurements might contribute to effectively follow‐up children particularly after surgery for UPJO. For this purpose, we retrospectively studied renal US reports of our patients who underwent pyeloplasty in the last 12 years and selected the ones only with normal contralateral kidneys to compare. As seen in Table 2, we found that kidneys with UPJO had a higher LL compared to contralaterals (99 mm vs. 85 mm, *p* < 0.0001) as expected but interestingly, not the TW (38 mm vs. 36 mm, *p* = 0.051). As noted in Table 1, pyeloplasty has decreased the LL of affected significantly (99 mm vs. 95 mm, *p* = 0.005) as RPAPD decreased more significantly (29 mm vs. 18 mm, *p* < 0.0001). However, TW remained stable following surgery (38 mm vs. 37 mm, *p* = 0.19). In our group, we have only 1 case requiring secondary intervention due to failed surgery and it is the only one with an increased LL at the 6th month US control. We can speculate that a decrease in LL of kidney would correlate to a decreased RPAPD to demonstrate the success of pyeloplasty. Table 3 shows that the strong correlation of RPAPD to LL of the kidney (correlation coefficient: 0.619, *p* < 0.001). Although we found a correlation of TW and hydronephrosis grade, we believe that it is a misleading finding since reports were written by different radiologists and there is no nominal standardization in classifying the hydronephrosis.

As the kidneys grow after birth, renal dimensions increase even if there is no change in RPAPD. Therefore, it is important to know how the kidney size changes. The LL of the kidney is approximately 4.5 cm in newborns. At the age of 1 year, LL reaches approximately 6 cm, and at the age of 10, it reaches approximately 9 cm in renal ultrasound [23]. Rosenbaum et al. [24] suggest the formula: length (cm) = 4.98 + (0.155 × age [months]) for the first year of life, and length (cm) = 6.79 + (0.22 × age [years]) for the children aged 1 year or older. Because of changing renal dimensions, it will be supportive and beneficial to use percentiles or graphs [23]. Kidney measurements can be examined according to the age of children; however, insufficient number of patients limits the sub‐analyses in our study. Therefore, we preferred to use the ratio of affected kidney to normal contralateral kidney dimensions in this study. As seen in Table 1, the ratio of LL of both kidneys reveals a significant decrease following surgery (1.2 vs. 1.12, *p* = 0.026), whereas the ratio of TW remains stable (1.15 vs. 1.06, *p* = 0.357). Therefore, we again speculate that we can utilize the change in the ratio of LL of the affected kidney to contralateral normal kidney would predict the success of pyeloplasty.

We also studied if the preoperative dimensions of kidney would correlate with diuretic response in nuclear renal dynamic scintigraphy with MAG‐3. As seen in Figures 2 and 3, LL and TW would not help to predict the diuretic response as well as renal pelvis diameter (Figure 1). Hence, we do not advocate the US‐measured dimensions of kidney to contribute for surgical indication decision making. However, although the LL of the affected kidney found still longer to contralateral normal kidney, the LL alone and ratio of LL of both kidneys significantly decrease postoperatively. We believe that in follow‐up of surgery for UPJO LL of kidney is a fast, undemanding, and reliable measurement to predict the success in the short term.

The present study has certain limitations. We do not routinely perform surgical renal pelvis reduction, but leave a ureteral stent for 6–8 weeks in pyeloplasty. We decide the success of pyeloplasty only with the resolution of pain and hydronephrosis. Nuclear dynamic renal scintigraphy with MAG‐3 is performed only in some cases where a decision cannot be made with renal US. Different technicians and radiologists performed and reported renal US. We could not reach some of the US images to confirm the measurements. We studied only the first renal US performed in the postoperative 6th month to predict the success of pyeloplasty. We point that long‐term follow‐up studies are required to establish LL as a reliable criterion for recurrence. Also, the absence of randomization and blinding may introduce bias in group allocation and outcome assessment.

In conclusion, we propose that LL may be used in conjunction with RPAPD to improve the reliability and reproducibility of sonographic evaluation.

## Author Contributions

Concept: D.D., O.C.O., C.A.S., S.Y. Design: D.D., O.C.O., C.A.S., S.Y. Data collection and processing: D.D., K.A.D., O.C.O. Statistical analysis: C.A.S. Data interpretation: D.D., C.A.S., Y.T., S.Y. Literature search: D.D., K.A.D. Writing: D.D., C.A.S., S.Y. Supervision: C.A.S., Y.T., S.Y., T.T.

## Ethics Statement

Ethical approval was obtained from the local ethics committee before the study (No: 09.2024.340).

## Consent

The authors have nothing to report.

## Conflicts of Interest

The authors declare no conflicts of interest.
